# Supplementary material for: Genome-wide analyses of the NAC transcription factor gene family in Acer palmatum provide valuable insights into the natural process of leaf senescence
Source: PeerJ. 2025 Jan 13;13:e18817. doi: 10.7717/peerj.18817 (PMC11737331; doi:10.7717/peerj.18817)
Supplement: Supplemental Information 1 [file peerj-13-18817-s001.docx]

Supplementary Table S1. Primer used for qRT-PCR analysis of *ApNACs*

| ApNAC | Primer pairs | |
| --- | --- | --- |
|  | Forward primer (5'-3') | Reverse primer (5'-3') |
| ApNAC06 | ACTGAAGCTGGTTATTGGAA | CAGTTGCTCTTTTCACCT |
| ApNAC41 | CAAACAGTCGAGAGAAGC | AATTCTTGCCTGGTCGTG |
| ApNAC91 | CTCAACCGCCTTATGGAC | CATTTGTTGTGAGCCGAT |
| ApNAC02 | CTTCCACTCCTTTCCCTC | TCCAATACCCAGATGCAG |
| ApNAC83 | ACTGGAAGTCTACCGGGA | GCATGACCCAATCCGTTC |
| ApNAC48 | CCTCTGCTGTCAAAACCA | ACACTCGACAGATTACCC |
| ApNAC51 | AGAAGGAATCAGAATCAGGT | TTGAAAAGTGGTGGTCGGAA |
| ApNAC04 | GATCTCAACAAGTGCGAACC | ACTGCCCTATCTTTTCCTGT |
| ApNAC05 | ATAAGCATGGTGGAGGCAAA | CATTCCTTCCCTCCAACG |
| ApNAC100 | AGTGTAAATGATCTCCCTCCTG | ATGCCTTCCCATTGAGTT |
| ApNAC80 | GATCGACCAGTCAAGGCCAA | AGGGTTTTTCTTGCTGGGGT |
| ApNAC116 | ATGTGGGACGTGGGAGAAAC | TCGAGATGGCGGACTCAATG |
| ApNAC15 | CTGATGACCCTCCTTCGCTC | TCACACGACCATCCCTGTTG |
| ApNAC79 | CCCTGCGGTTCCTAATCCAT | TTTGCGACCGAAGGGAAGAG |
| ApNAC76 | AGGTACAACCTCCGGTTCCT | GGCGACTGAAAGGTGGACAA |
| actin | CATGATTGGAATGGAAGCTG | TTCCTTGCTCATTCTGTCAG |
