# Supplementary material for: Genome-wide analyses of the NAC transcription factor gene family in Acer palmatum provide valuable insights into the natural process of leaf senescence
Source: PeerJ. 2025 Jan 13;13:e18817. doi: 10.7717/peerj.18817 (PMC11737331; doi:10.7717/peerj.18817)
Supplement: Supplemental Information 3 [file peerj-13-18817-s003.docx]

Supplementary Table S3. Annotation of *A. palmatum* NAC transcription factors

| Name | Chromesome | Gene ID | Amino acid length / aa | Relative molecular weight / kDa | Theoretical isoelectric point (pI) | Subcellular localization |
| --- | --- | --- | --- | --- | --- | --- |
| ApNAC01 | Chr01 | A.palmatum_LG01_G01016.t1 | 212 | 24276.61 | 5.24 | Nucleus |
| ApNAC02 | Chr01 | A.palmatum_LG01_G01055.t1 | 381 | 43454.55 | 7.35 | Nucleus |
| ApNAC03 | Chr01 | A.palmatum_LG01_G01058.t1 | 396 | 44792.61 | 5.79 | Nucleus |
| ApNAC04 | Chr01 | A.palmatum_LG01_G01429.t1 | 318 | 35733.19 | 6.82 | Nucleus |
| ApNAC05 | Chr01 | A.palmatum_LG01_G01431.t1 | 318 | 35718.21 | 6.82 | Nucleus |
| ApNAC06 | Chr01 | A.palmatum_LG01_G01723.t1 | 379 | 41395.16 | 8.56 | Nucleus |
| ApNAC07 | Chr01 | A.palmatum_LG01_G01958.t1 | 340 | 38174.69 | 8.20 | Nucleus |
| ApNAC08 | Chr01 | A.palmatum_LG01_G01962.t1 | 371 | 41280.39 | 7.80 | Nucleus |
| ApNAC09 | Chr02 | A.palmatum_LG02_G00653.t1 | 289 | 33156.56 | 8.17 | Nucleus |
| ApNAC10 | Chr02 | A.palmatum_LG02_G00950.t1 | 223 | 25436.22 | 6.61 | Nucleus |
| ApNAC11 | Chr02 | A.palmatum_LG02_G00973.t1 | 390 | 43150.28 | 5.71 | Nucleus |
| ApNAC12 | Chr02 | A.palmatum_LG02_G01744.t1 | 255 | 30217.26 | 5.84 | Nucleus |
| ApNAC13 | Chr02 | A.palmatum_LG02_G01745.t1 | 216 | 25166.53 | 5.82 | Nucleus |
| ApNAC14 | Chr03 | A.palmatum_LG03_G00309.t1 | 225 | 26088.38 | 9.06 | Nucleus |
| ApNAC15 | Chr03 | A.palmatum_LG03_G00857.t1 | 375 | 42514.9 | 8.39 | Nucleus |
| ApNAC16 | Chr03 | A.palmatum_LG03_G00867.t1 | 593 | 66408.85 | 5.83 | Nucleus |
| ApNAC17 | Chr03 | A.palmatum_LG03_G00869.t1 | 345 | 38901.93 | 5.85 | Nucleus |
| ApNAC18 | Chr03 | A.palmatum_LG03_G01645.t1 | 648 | 74384.84 | 5.33 | Nucleus |
| ApNAC19 | Chr03 | A.palmatum_LG03_G01819.t1 | 242 | 27916.3 | 8.85 | Nucleus |
| ApNAC20 | Chr03 | A.palmatum_LG03_G01848.t1 | 241 | 27857.3 | 8.85 | Nucleus |
| ApNAC21 | Chr03 | A.palmatum_LG03_G02185.t1 | 297 | 33565.21 | 5.65 | Nucleus |
| ApNAC22 | Chr03 | A.palmatum_LG03_G02186.t1 | 575 | 65473.37 | 5.74 | Nucleus |
| ApNAC23 | Chr03 | A.palmatum_LG03_G02190.t1 | 286 | 32657.23 | 5.20 | Nucleus |
| ApNAC24 | Chr03 | A.palmatum_LG03_G02192.t1 | 274 | 29927.78 | 9.28 | Nucleus |
| ApNAC25 | Chr03 | A.palmatum_LG03_G02263.t1 | 174 | 20243.87 | 5.86 | Nucleus |
| ApNAC26 | Chr03 | A.palmatum_LG03_G02321.t1 | 237 | 27545.52 | 7.70 | Nucleus |
| ApNAC27 | Chr03 | A.palmatum_LG03_G02322.t1 | 216 | 24739.32 | 8.70 | Nucleus |
| ApNAC28 | Chr03 | A.palmatum_LG03_G02570.t1 | 139 | 15833.98 | 6.59 | Nucleus |
| ApNAC29 | Chr03 | A.palmatum_LG03_G02861.t1 | 344 | 39565.32 | 8.79 | Nucleus |
| ApNAC30 | Chr03 | A.palmatum_LG03_G02919.t1 | 334 | 38098.86 | 7.30 | Nucleus |
| ApNAC31 | Chr03 | A.palmatum_LG03_G02949.t1 | 343 | 39248.6 | 4.99 | Nucleus |
| ApNAC32 | Chr03 | A.palmatum_LG03_G02963.t1 | 214 | 24196.16 | 5.47 | Cytoplasm |
| ApNAC33 | Chr03 | A.palmatum_LG03_G02964.t1 | 341 | 39014.64 | 5.02 | Cytoplasm |
| ApNAC34 | Chr03 | A.palmatum_LG03_G02982.t1 | 501 | 55401.94 | 6.34 | Cytoplasm |
| ApNAC35 | Chr04 | A.palmatum_LG04_G00709.t1 | 396 | 44823.54 | 6.34 | Nucleus |
| ApNAC36 | Chr04 | A.palmatum_LG04_G00742.t1 | 380 | 43717.38 | 6.37 | Nucleus |
| ApNAC37 | Chr04 | A.palmatum_LG04_G00743.t1 | 221 | 25933.62 | 6.44 | Nucleus |
| ApNAC38 | Chr04 | A.palmatum_LG04_G00744.t1 | 467 | 54273.98 | 7.25 | Cytoplasm |
| ApNAC39 | Chr04 | A.palmatum_LG04_G01412.t1 | 228 | 25653.69 | 5.67 | Nucleus |
| ApNAC40 | Chr04 | A.palmatum_LG04_G02552.t1 | 206 | 23657.25 | 8.83 | Cytoplasm |
| ApNAC41 | Chr05 | A.palmatum_LG05_G00690.t1 | 312 | 34681.72 | 8.91 | Nucleus |
| ApNAC42 | Chr05 | A.palmatum_LG05_G00886.t1 | 249 | 28092 | 9.51 | Chloroplast |
| ApNAC43 | Chr05 | A.palmatum_LG05_G01068.t1 | 390 | 43886.72 | 5.63 | Nucleus |
| ApNAC44 | Chr05 | A.palmatum_LG05_G01285.t1 | 306 | 35576.04 | 8.84 | Nucleus |
| ApNAC45 | Chr05 | A.palmatum_LG05_G01415.t1 | 238 | 27346.69 | 8.74 | Nucleus |
| ApNAC46 | Chr05 | A.palmatum_LG05_G01501.t1 | 241 | 27236.13 | 6.12 | Nucleus |
| ApNAC47 | Chr05 | A.palmatum_LG05_G01516.t1 | 369 | 41562.31 | 5.00 | Nucleus |
| ApNAC48 | Chr05 | A.palmatum_LG05_G02035.t1 | 198 | 22748.34 | 5.10 | Cytoplasm |
| ApNAC49 | Chr05 | A.palmatum_LG05_G02286.t1 | 404 | 45951.98 | 6.86 | Nucleus |
| ApNAC50 | Chr05 | A.palmatum_LG05_G02318.t1 | 279 | 30367.42 | 10.02 | Chloroplast |
| ApNAC51 | Chr06 | A.palmatum_LG06_G00254.t1 | 295 | 34206.91 | 6.20 | Nucleus |
| ApNAC52 | Chr06 | A.palmatum_LG06_G00320.t1 | 482 | 54102.53 | 5.46 | Nucleus |
| ApNAC53 | Chr06 | A.palmatum_LG06_G00833.t1 | 476 | 53901.6 | 6.55 | Nucleus |
| ApNAC54 | Chr06 | A.palmatum_LG06_G01629.t1 | 387 | 43932.46 | 4.50 | Nucleus |
| ApNAC55 | Chr06 | A.palmatum_LG06_G01630.t1 | 384 | 43653.8 | 5.02 | Nucleus |
| ApNAC56 | Chr06 | A.palmatum_LG06_G01631.t1 | 560 | 64104.11 | 5.42 | Cytoplasm |
| ApNAC57 | Chr06 | A.palmatum_LG06_G01633.t1 | 544 | 62755.63 | 5.27 | Cytoplasm |
| ApNAC58 | Chr06 | A.palmatum_LG06_G01634.t1 | 225 | 25951.13 | 5.01 | Nucleus |
| ApNAC59 | Chr06 | A.palmatum_LG06_G01801.t1 | 351 | 38908.71 | 8.87 | Nucleus |
| ApNAC60 | Chr07 | A.palmatum_LG07_G00206.t1 | 181 | 20363.61 | 9.65 | Cytoplasm |
| ApNAC61 | Chr07 | A.palmatum_LG07_G00209.t1 | 157 | 18230.63 | 7.82 | Cytoplasm |
| ApNAC62 | Chr07 | A.palmatum_LG07_G00311.t1 | 284 | 31846.6 | 9.25 | Nucleus |
| ApNAC63 | Chr07 | A.palmatum_LG07_G00867.t1 | 445 | 50219.23 | 7.31 | Nucleus |
| ApNAC64 | Chr07 | A.palmatum_LG07_G00900.t1 | 298 | 34887.53 | 8.80 | Cytoplasm |
| ApNAC65 | Chr07 | A.palmatum_LG07_G00973.t1 | 659 | 73643.66 | 4.69 | Nucleus |
| ApNAC66 | Chr07 | A.palmatum_LG07_G00975.t1 | 172 | 19982.82 | 7.71 | Nucleus |
| ApNAC67 | Chr07 | A.palmatum_LG07_G01172.t1 | 425 | 47993.42 | 4.94 | Nucleus |
| ApNAC68 | Chr08 | A.palmatum_LG08_G00070.t1 | 394 | 44808.41 | 5.58 | Nucleus |
| ApNAC69 | Chr08 | A.palmatum_LG08_G00446.t1 | 696 | 77539.5 | 5.04 | Nucleus |
| ApNAC70 | Chr08 | A.palmatum_LG08_G00537.t1 | 194 | 22060.9 | 4.95 | Cytoplasm |
| ApNAC71 | Chr08 | A.palmatum_LG08_G00638.t1 | 290 | 32776.68 | 5.92 | Cytoplasm |
| ApNAC72 | Chr08 | A.palmatum_LG08_G01261.t1 | 303 | 34469.92 | 6.02 | Nucleus |
| ApNAC73 | Chr08 | A.palmatum_LG08_G01388.t1 | 324 | 37648.84 | 5.72 | Nucleus |
| ApNAC74 | Chr08 | A.palmatum_LG08_G01474.t1 | 603 | 68486.36 | 4.65 | Nucleus |
| ApNAC75 | Chr08 | A.palmatum_LG08_G01761.t1 | 306 | 35269.44 | 8.79 | Nucleus |
| ApNAC76 | Chr09 | A.palmatum_LG09_G00241.t1 | 613 | 68198.21 | 5.70 | Nucleus |
| ApNAC77 | Chr09 | A.palmatum_LG09_G00242.t1 | 367 | 41762.12 | 4.85 | Extracellular space |
| ApNAC78 | Chr09 | A.palmatum_LG09_G00243.t1 | 611 | 68030.67 | 5.50 | Nucleus |
| ApNAC79 | Chr09 | A.palmatum_LG09_G00244.t1 | 554 | 61896.13 | 5.96 | Nucleus |
| ApNAC80 | Chr09 | A.palmatum_LG09_G00245.t1 | 263 | 30456.15 | 5.82 | Nucleus |
| ApNAC81 | Chr09 | A.palmatum_LG09_G00668.t1 | 430 | 49001.2 | 6.33 | Nucleus |
| ApNAC82 | Chr09 | A.palmatum_LG09_G00690.t1 | 655 | 73862.13 | 5.04 | Nucleus |
| ApNAC83 | Chr09 | A.palmatum_LG09_G00841.t1 | 266 | 30650.9 | 5.26 | Nucleus |
| ApNAC84 | Chr09 | A.palmatum_LG09_G00998.t1 | 376 | 43123.06 | 6.64 | Nucleus |
| ApNAC85 | Chr09 | A.palmatum_LG09_G01330.t1 | 331 | 37395.03 | 7.65 | Nucleus |
| ApNAC86 | Chr09 | A.palmatum_LG09_G01646.t1 | 296 | 33515.29 | 6.95 | Nucleus |
| ApNAC87 | Chr10 | A.palmatum_LG10_G00728.t1 | 360 | 41120.15 | 7.67 | Nucleus |
| ApNAC88 | Chr10 | A.palmatum_LG10_G00972.t1 | 350 | 39820.23 | 5.12 | Nucleus |
| ApNAC89 | Chr10 | A.palmatum_LG10_G00974.t1 | 428 | 47974.81 | 4.66 | Nucleus |
| ApNAC90 | Chr10 | A.palmatum_LG10_G01039.t1 | 272 | 31240.79 | 6.08 | Nucleus |
| ApNAC91 | Chr10 | A.palmatum_LG10_G01040.t1 | 274 | 31344.95 | 6.37 | Nucleus |
| ApNAC92 | Chr10 | A.palmatum_LG10_G01657.t1 | 345 | 39179.07 | 5.65 | Nucleus |
| ApNAC93 | Chr10 | A.palmatum_LG10_G01765.t1 | 394 | 44549.3 | 6.27 | Nucleus |
| ApNAC94 | Chr11 | A.palmatum_LG11_G00658.t1 | 335 | 38687.03 | 5.37 | Nucleus |
| ApNAC95 | Chr11 | A.palmatum_LG11_G00664.t1 | 238 | 26718.28 | 8.26 | Nucleus |
| ApNAC96 | Chr11 | A.palmatum_LG11_G01137.t1 | 294 | 33763.42 | 6.12 | Nucleus |
| ApNAC97 | Chr11 | A.palmatum_LG11_G01203.t1 | 345 | 38814.63 | 4.65 | Cytoplasm |
| ApNAC98 | Chr11 | A.palmatum_LG11_G01204.t1 | 340 | 38303.97 | 4.61 | Cytoplasm |
| ApNAC99 | Chr11 | A.palmatum_LG11_G01206.t1 | 345 | 38746.79 | 4.72 | Cytoplasm |
| ApNAC100 | Chr11 | A.palmatum_LG11_G01245.t1 | 218 | 25182.2 | 4.49 | Nucleus |
| ApNAC101 | Chr12 | A.palmatum_LG12_G01027.t1 | 325 | 36391.83 | 6.37 | Nucleus |
| ApNAC102 | Chr12 | A.palmatum_LG12_G01029.t1 | 325 | 36565.6 | 6.43 | Nucleus |
| ApNAC103 | Chr12 | A.palmatum_LG12_G01177.t1 | 410 | 45577.49 | 8.18 | Nucleus |
| ApNAC104 | Chr13 | A.palmatum_LG13_G00023.t1 | 620 | 69253.71 | 4.87 | Nucleus |
| ApNAC105 | Chr13 | A.palmatum_LG13_G00560.t1 | 341 | 38260.8 | 5.65 | Nucleus |
| ApNAC106 | Chr13 | A.palmatum_LG13_G00561.t1 | 422 | 47964.38 | 4.72 | Nucleus |
| ApNAC107 | Chr13 | A.palmatum_LG13_G00605.t1 | 620 | 70659.01 | 5.33 | Nucleus |
| ApNAC108 | Chr13 | A.palmatum_LG13_G00606.t1 | 871 | 98111.96 | 5.31 | Nucleus |
| ApNAC109 | Chr13 | A.palmatum_LG13_G00627.t1 | 377 | 42957.94 | 4.99 | Nucleus |
| ApNAC110 | Chr13 | A.palmatum_LG13_G00629.t1 | 437 | 49588.34 | 4.88 | Nucleus |
| ApNAC111 | Chr13 | A.palmatum_LG13_G00630.t1 | 311 | 35520.7 | 5.18 | Nucleus |
| ApNAC112 | Chr13 | A.palmatum_LG13_G00676.t1 | 694 | 79854.04 | 6.77 | Nucleus |
| ApNAC113 | Chr13 | A.palmatum_LG13_G00705.t1 | 211 | 24851.27 | 9.34 | Nucleus |
| ApNAC114 | Chr13 | A.palmatum_LG13_G01081.t1 | 449 | 49751.98 | 4.22 | Chloroplast |
| ApNAC115 | Chr13 | A.palmatum_LG13_G01082.t1 | 423 | 47776.31 | 5.58 | Chloroplast |
| ApNAC116 | Chr13 | A.palmatum_LG13_G01572.t1 | 374 | 40790.63 | 5.97 | Cytoplasm |
