# Supplementary material for: Genome-wide analyses of the NAC transcription factor gene family in Acer palmatum provide valuable insights into the natural process of leaf senescence
Source: PeerJ. 2025 Jan 13;13:e18817. doi: 10.7717/peerj.18817 (PMC11737331; doi:10.7717/peerj.18817)
Supplement: Supplemental Information 4 [file peerj-13-18817-s004.docx]

Supplementary Table S4. Details of gene segmental-duplication of *ApNACs*

| Gene1 | Chromsome | Localization | Gene2 | Chromsome | Localization | Duplication |
| --- | --- | --- | --- | --- | --- | --- |
| ApNAC07 | LG01 | 1952420-1954549 | ApNAC103 | LG12 | 33615457-33616956 | Segmental |
| ApNAC09 | LG02 | 12271496-12273886 | ApNAC87 | LG10 | 9628111-9630105 | Segmental |
| ApNAC10 | LG02 | 1575855-1576587 | ApNAC90 | LG10 | 15052956-15054909 | Segmental |
| ApNAC35 | LG04 | 2845984-2847971 | ApNAC81 | LG09 | 7789578-7791094 | Segmental |
| ApNAC36 | LG04 | 2845984-2847971 | ApNAC82 | LG09 | 7949795-7952660 | Segmental |
| ApNAC41 | LG05 | 4257070-4258248 | ApNAC102 | LG05 | 20432586-20433385 | Segmental |
| ApNAC42 | LG05 | 4257070-4258248 | ApNAC45 | LG07 | 3828842-3829914 | Segmental |
| ApNAC01 | LG05 | 8571932-8575841 | ApNAC62 | LG12 | 31532079-31534519 | Segmental |
| ApNAC72 | LG08 | 10268576-10273325 | ApNAC96 | LG11 | 21110103-21111183 | Segmental |
| ApNAC86 | LG09 | 4833704-4835719 | ApNAC92 | LG10 | 39396952-39398180 | Segmental |
