# Supplementary material for: Genome-wide analyses of the NAC transcription factor gene family in Acer palmatum provide valuable insights into the natural process of leaf senescence
Source: PeerJ. 2025 Jan 13;13:e18817. doi: 10.7717/peerj.18817 (PMC11737331; doi:10.7717/peerj.18817)
Supplement: Supplemental Information 5 [file peerj-13-18817-s005.docx]

Supplementary Table S5. Details of gene tandem-duplication of *ApNACs*

| Gene | Chromsome | Localization | Duplication |
| --- | --- | --- | --- |
| ApNAC12 | Chr02 | 50605865-50609267 | Tandem |
| ApNAC13 | Chr02 | 50737450-50738600 | Tandem |
| ApNAC21 | Chr03 | 61695538-61696723 | Tandem |
| ApNAC22 | Chr03 | 61728211-61738151 | Tandem |
| ApNAC26 | Chr03 | 64556692-64557598 | Tandem |
| ApNAC27 | Chr03 | 64565520-64566337 | Tandem |
| ApNAC32 | Chr03 | 75636643-75637531 | Tandem |
| ApNAC33 | Chr03 | 75639488-75644977 | Tandem |
| ApNAC36 | Chr04 | 10215738-10217970 | Tandem |
| ApNAC37 | Chr04 | 10224044-10253482 | Tandem |
| ApNAC38 | Chr04 | 10273683-10278177 | Tandem |
| ApNAC54 | Chr06 | 45774133-45775896 | Tandem |
| ApNAC55 | Chr06 | 45779719-45782133 | Tandem |
| ApNAC56 | Chr06 | 45789905-45792324 | Tandem |
| ApNAC57 | Chr06 | 45818753-45821101 | Tandem |
| ApNAC58 | Chr06 | 45826186-45827062 | Tandem |
| ApNAC65 | Chr07 | 12606947-12615166 | Tandem |
| ApNAC66 | Chr07 | 12619827-12620571 | Tandem |
| ApNAC76 | Chr09 | 2766859-2779917 | Tandem |
| ApNAC77 | Chr09 | 2789563-2823044 | Tandem |
| ApNAC78 | Chr09 | 2824766-2828314 | Tandem |
| ApNAC79 | Chr09 | 2848768-2877736 | Tandem |
| ApNAC80 | Chr09 | 2896407-2946812 | Tandem |
| ApNAC88 | Chr10 | 14043558-14044610 | Tandem |
| ApNAC89 | Chr10 | 14060897-14067767 | Tandem |
| ApNAC90 | Chr10 | 15052956-15054909 | Tandem |
| ApNAC91 | Chr10 | 15107487-15109235 | Tandem |
| ApNAC97 | Chr11 | 23160603-23163692 | Tandem |
| ApNAC98 | Chr11 | 23202633-23206763 | Tandem |
| ApNAC105 | Chr12 | 18837304-18840087 | Tandem |
| ApNAC106 | Chr12 | 18854619-18857397 | Tandem |
| ApNAC107 | Chr12 | 19765359-19769710 | Tandem |
| ApNAC108 | Chr12 | 19771224-19784320 | Tandem |
| ApNAC110 | Chr12 | 20141184-20145851 | Tandem |
| ApNAC111 | Chr12 | 20146708-20147847 | Tandem |
| ApNAC114 | Chr12 | 28828878-28831432 | Tandem |
| ApNAC115 | Chr12 | 28834988-28839312 | Tandem |
