# Supplementary material for: Genome-wide analyses of the NAC transcription factor gene family in Acer palmatum provide valuable insights into the natural process of leaf senescence
Source: PeerJ. 2025 Jan 13;13:e18817. doi: 10.7717/peerj.18817 (PMC11737331; doi:10.7717/peerj.18817)
Supplement: Supplemental Information 7 [file peerj-13-18817-s007.docx]

Supplementary Table S7. Expression patterns of 68 *ApNACs* during *A. palmatum* leaf senescense

| Gene | AUS-1_fpkm | AUS-2_fpkm | AUS-3_fpkm | SUS-1_fpkm | SUS-2_fpkm | SUS-3_fpkm |
| --- | --- | --- | --- | --- | --- | --- |
| ApNAC02 | 9.629648044 | 9.460005304 | 9.65901474 | 1.416839742 | 1.097610797 | 1.244887059 |
| ApNAC03 | 7.25502857 | 7.370164281 | 7.214999019 | 4.693765712 | 3.757023247 | 3.870857864 |
| ApNAC04 | 8.449973986 | 8.657782896 | 8.348329809 | 1.531069493 | 2.250961574 | 1.867896464 |
| ApNAC05 | 8.274261661 | 8.546508661 | 8.176073391 | 2.073820233 | 1.589763487 | 1.867896464 |
| ApNAC06 | 3.125981654 | 2.111031312 | 2.381283373 | 0 | 0 | 0 |
| ApNAC07 | 10.02472414 | 10.21386973 | 10.088404 | 3.912649865 | 4.105175192 | 3.63691458 |
| ApNAC08 | 9.546064865 | 9.535489237 | 9.599726916 | 3.754887502 | 3.882643049 | 3.534808661 |
| ApNAC10 | 2.961623328 | 2.901108243 | 2.482848283 | 2.166715445 | 2.192194165 | 2.748461233 |
| ApNAC11 | 4.181102551 | 4.247927513 | 4.183486514 | 2.09085343 | 2.97819563 | 2.657640005 |
| ApNAC15 | 0 | 0 | 0 | 0.070389328 | 0.084064265 | 0.070389328 |
| ApNAC18 | 0.695993813 | 0.443606651 | 0.50589093 | 0.176322773 | 0.201633861 | 0.042644337 |
| ApNAC24 | 0 | 0.111031312 | 0.111031312 | 0.5360529 | 0 | 0.097610797 |
| ApNAC26 | 3.224966365 | 3.161887682 | 2.776103988 | 4.253232939 | 3.111031312 | 3.073820233 |
| ApNAC29 | 3.595742339 | 4.29130886 | 3.720278465 | 1.925999419 | 1.819668183 | 1.843983844 |
| ApNAC30 | 2.333423734 | 2.572889668 | 2.269033146 | 2.319039816 | 1.989139007 | 1.803227036 |
| ApNAC38 | 0.443606651 | 0.575312331 | 0.475084883 | 0.613531653 | 0.831877241 | 0.545968369 |
| ApNAC39 | 1.599317794 | 1.835924074 | 2.403267722 | 0.5360529 | 0.275007047 | 0 |
| ApNAC40 | 0.86393845 | 0.378511623 | 0.526068812 | 1.580145484 | 1.117695043 | 1.967168608 |
| ApNAC41 | 8.189873965 | 8.69926011 | 8.157195331 | 0.263034406 | 0.111031312 | 0.454175893 |
| ApNAC42 | 6.3305584 | 6.534341795 | 6.018589668 | 4.027684877 | 3.032100843 | 3.559491813 |
| ApNAC43 | 0.286881148 | 0 | 0.163498732 | 1.90303827 | 1.084064265 | 0.956056652 |
| ApNAC44 | 3.930737338 | 4.083213368 | 3.539779192 | 3.913607512 | 2.807354922 | 3.058316496 |
| ApNAC47 | 0.545968369 | 0.695993813 | 0.575312331 | 2.080657663 | 1.207892852 | 1.655351829 |
| ApNAC48 | 0 | 0.40053793 | 0.150559677 | 0 | 0 | 0 |
| ApNAC49 | 4.062639828 | 4.085764554 | 3.882643049 | 4.777156666 | 5.019257119 | 4.969012308 |
| ApNAC51 | 0.275007047 | 0.097610797 | 0.201633861 | 0 | 0 | 0 |
| ApNAC52 | 0.23878686 | 0 | 0.189033824 | 1.077242999 | 0.847996907 | 0.443606651 |
| ApNAC53 | 2.757023247 | 3.107687869 | 3.145677455 | 3.414135533 | 3.099295204 | 2.778208576 |
| ApNAC55 | 1.89917563 | 1.622930351 | 1.195347598 | 0 | 0 | 0.443606651 |
| ApNAC56 | 2.185866545 | 2.039138394 | 1.752748591 | 0.526068812 | 0.378511623 | 0.632268215 |
| ApNAC59 | 0.731183242 | 0.879705766 | 0.933572638 | 0.23878686 | 0.097610797 | 0.687060688 |
| ApNAC62 | 7.015582312 | 7.072427412 | 6.702934532 | 5.788946667 | 5.889230152 | 5.321928095 |
| ApNAC64 | 10.1883663 | 10.44714523 | 9.934133132 | 4.355439197 | 3.932628157 | 3.458119481 |
| ApNAC65 | 4.478971805 | 4.391630262 | 4.415488271 | 4.084064265 | 3.90303827 | 3.619413011 |
| ApNAC66 | 0 | 0.163498732 | 0.176322773 | 1.13093087 | 1.344828497 | 0.286881148 |
| ApNAC67 | 3.23878686 | 3.260025656 | 2.965322548 | 3.077242999 | 3.070389328 | 3.270528942 |
| ApNAC69 | 5.173527238 | 5.241840184 | 5.429281193 | 5.122258568 | 4.482848283 | 5.040015679 |
| ApNAC71 | 2.956056652 | 2.292781749 | 2.66448284 | 4.91981677 | 4.965322548 | 5.023698917 |
| ApNAC72 | 8.418738108 | 8.543805176 | 8.599354991 | 7.387328072 | 7.057666877 | 7.116655862 |
| ApNAC73 | 1.427606173 | 1.618238656 | 0.98550043 | 0.176322773 | 0.097610797 | 0.084064265 |
| ApNAC74 | 7.672566776 | 7.811085644 | 7.679268958 | 6.437960088 | 6.451870479 | 6.508745554 |
| ApNAC76 | 0.097610797 | 0.275007047 | 0.150559677 | 1.40053793 | 1.40053793 | 1.967168608 |
| ApNAC77 | 2.438292852 | 2.386810946 | 2.482848283 | 5.730911624 | 5.365972428 | 5.587364991 |
| ApNAC78 | 1.782408565 | 1.422233001 | 1.827819025 | 5.338780944 | 4.906890596 | 4.926948248 |
| ApNAC79 | 0.941106311 | 0.250961574 | 0.782408565 | 4.581351247 | 4.146492307 | 4.580145484 |
| ApNAC80 | 0 | 0 | 0 | 2.147306699 | 2.073820233 | 2.153805336 |
| ApNAC82 | 6.223615617 | 6.270528942 | 6.345715709 | 5.007644031 | 5.111031312 | 4.834407842 |
| ApNAC83 | 2.827819025 | 3.174725988 | 3.106013238 | 0.111031312 | 0 | 0 |
| ApNAC84 | 0.584962501 | 1.389566812 | 0.739848103 | 0.070389328 | 0.084064265 | 0.070389328 |
| ApNAC85 | 2.881664619 | 2.72900887 | 2.965322548 | 0.748461233 | 0.201633861 | 0.432959407 |
| ApNAC86 | 7.08841725 | 7.108106224 | 7.264348763 | 4.253989266 | 4.257010618 | 4.40053793 |
| ApNAC90 | 0.495695163 | 0.757023247 | 0.545968369 | 0 | 0 | 0.773996325 |
| ApNAC91 | 1.03562391 | 0.545968369 | 0.454175893 | 0 | 0 | 0 |
| ApNAC92 | 3.929790998 | 3.787641414 | 3.50589093 | 1.316145742 | 0.933572638 | 1.794935663 |
| ApNAC93 | 0.275007047 | 0.150559677 | 1.111031312 | 1.269033146 | 1.411426246 | 0.910732662 |
| ApNAC95 | 0.526068812 | 0.695993813 | 0.895302621 | 0 | 0 | 0.111031312 |
| ApNAC96 | 5.145677455 | 5.773468928 | 5.488000771 | 3.715893371 | 3.49441561 | 4.336283388 |
| ApNAC97 | 4.575312331 | 4.09423607 | 4.49441561 | 5.241077458 | 5.060479781 | 5.049195106 |
| ApNAC98 | 5.723012396 | 5.544114402 | 5.840714991 | 5.346956889 | 5.140369671 | 4.971773447 |
| ApNAC99 | 4.832890014 | 4.803227036 | 5.017031081 | 4.44625623 | 4.054848477 | 3.97819563 |
| ApNAC100 | 0 | 0.137503524 | 0.275007047 | 0 | 0 | 0 |
| ApNAC102 | 6.179112901 | 6.332707934 | 6.156032499 | 5.619119511 | 4.776103988 | 4.498250868 |
| ApNAC103 | 5.393690764 | 4.94016675 | 5.207111961 | 3.884597921 | 4.286881148 | 4.264536431 |
| ApNAC104 | 5.199279721 | 5.165107985 | 4.993221467 | 5.651625784 | 4.910732662 | 4.994127114 |
| ApNAC112 | 1.794935663 | 1.531069493 | 1.3950628 | 2.307428525 | 2.341985747 | 2.3950628 |
| ApNAC114 | 7.917909074 | 7.964514154 | 7.973152038 | 6.13093087 | 5.927185358 | 5.604071324 |
| ApNAC115 | 5.205548911 | 5.17512535 | 5.220716892 | 5.245267467 | 4.906409617 | 4.907371414 |
| ApNAC116 | 0 | 0 | 0 | 0.411426246 | 0.084064265 | 0 |
