# Supplementary material for: Genome-wide analyses of the NAC transcription factor gene family in Acer palmatum provide valuable insights into the natural process of leaf senescence
Source: PeerJ. 2025 Jan 13;13:e18817. doi: 10.7717/peerj.18817 (PMC11737331; doi:10.7717/peerj.18817)
Supplement: Supplemental Information 8 [file peerj-13-18817-s008.docx]

Supplementary Table S8.Coexpression network analysis between 10 *ApNACs* and other genes

| TFs | NAC | COR |
| --- | --- | --- |
| MYB_related16 | ApNAC91 | 0.9994854 |
| ERF80 | ApNAC91 | 0.9994156 |
| MYB_related4 | ApNAC91 | 0.9992638 |
| ERF57 | ApNAC91 | 0.9984134 |
| WRKY15 | ApNAC91 | 0.9983283 |
| ERF54 | ApNAC91 | 0.9981686 |
| bZIP31 | ApNAC91 | 0.99685 |
| ERF74 | ApNAC91 | 0.9959772 |
| bZIP4 | ApNAC91 | 0.9959489 |
| ERF8 | ApNAC91 | 0.9958198 |
| C2H22 | ApNAC91 | 0.9954915 |
| WRKY4 | ApNAC91 | 0.9951771 |
| ERF96 | ApNAC91 | 0.9950275 |
| MYB_related56 | ApNAC91 | 0.9944484 |
| WRKY3 | ApNAC91 | 0.9940422 |
| MYB_related1 | ApNAC91 | 0.9935838 |
| MYB_related5 | ApNAC91 | 0.9935114 |
| bZIP35 | ApNAC91 | 0.9933014 |
| bZIP26 | ApNAC91 | 0.9932448 |
| WRKY22 | ApNAC91 | 0.9932129 |
| bZIP43 | ApNAC91 | 0.9930812 |
| ERF97 | ApNAC91 | 0.9930213 |
| ERF19 | ApNAC91 | 0.9926892 |
| bZIP37 | ApNAC91 | 0.9921922 |
| bZIP5 | ApNAC91 | 0.9917224 |
| ERF11 | ApNAC91 | 0.9917053 |
| MYB_related48 | ApNAC91 | 0.9912942 |
| ERF77 | ApNAC91 | 0.9907851 |
| bZIP8 | ApNAC91 | 0.9904485 |
| WRKY | ApNAC91 | 0.990202 |
| C2H28 | ApNAC02 | 0.9999857 |
| WRKY29 | ApNAC02 | 0.9996199 |
| bZIP14 | ApNAC02 | 0.9995349 |
| bZIP13 | ApNAC02 | 0.9989994 |
| WRKY49 | ApNAC02 | 0.9988939 |
| MYB_related41 | ApNAC02 | 0.9988438 |
| ERF58 | ApNAC02 | 0.998798 |
| WRKY50 | ApNAC02 | 0.9985813 |
| WRKY40 | ApNAC02 | 0.9983804 |
| WRKY34 | ApNAC02 | 0.9983727 |
| WRKY31 | ApNAC02 | 0.9980933 |
| WRKY5 | ApNAC02 | 0.9978565 |
| bZIP12 | ApNAC02 | 0.9978333 |
| WRKY20 | ApNAC02 | 0.9970731 |
| ERF4 | ApNAC02 | 0.9969415 |
| bZIP10 | ApNAC02 | 0.9961674 |
| MYB_related12 | ApNAC02 | 0.9958871 |
| MYB_related10 | ApNAC02 | 0.9954584 |
| ERF11 | ApNAC02 | 0.9953747 |
| MYB_related1 | ApNAC02 | 0.9947141 |
| ERF96 | ApNAC02 | 0.9936606 |
| C2H213 | ApNAC02 | 0.99353 |
| bZIP37 | ApNAC02 | 0.9931741 |
| WRKY32 | ApNAC02 | 0.9923017 |
| C2H22 | ApNAC02 | 0.9921132 |
| ERF19 | ApNAC02 | 0.9915495 |
| bZIP14 | ApNAC83 | 0.9996407 |
| C2H28 | ApNAC83 | 0.9994262 |
| WRKY29 | ApNAC83 | 0.9983368 |
| bZIP12 | ApNAC83 | 0.99824 |
| WRKY40 | ApNAC83 | 0.9980819 |
| ERF58 | ApNAC83 | 0.9980434 |
| WRKY34 | ApNAC83 | 0.9978608 |
| WRKY49 | ApNAC83 | 0.9974774 |
| bZIP13 | ApNAC83 | 0.9973915 |
| MYB_related41 | ApNAC83 | 0.9972799 |
| WRKY50 | ApNAC83 | 0.996572 |
| WRKY5 | ApNAC83 | 0.9961478 |
| ERF4 | ApNAC83 | 0.9960419 |
| WRKY31 | ApNAC83 | 0.9960125 |
| WRKY20 | ApNAC83 | 0.9943458 |
| bZIP10 | ApNAC83 | 0.9942703 |
| MYB_related12 | ApNAC83 | 0.9939228 |
| ERF11 | ApNAC83 | 0.9926555 |
| MYB_related10 | ApNAC83 | 0.9924055 |
| C2H213 | ApNAC83 | 0.9923866 |
| MYB_related1 | ApNAC83 | 0.9915169 |
| WRKY32 | ApNAC83 | 0.9914677 |
| WRKY30 | ApNAC83 | 0.9912887 |
| bZIP37 | ApNAC83 | 0.990214 |
| WRKY5 | ApNAC41 | 0.999861 |
| bZIP10 | ApNAC41 | 0.9997224 |
| ERF58 | ApNAC41 | 0.9992615 |
| ERF11 | ApNAC41 | 0.9992115 |
| MYB_related1 | ApNAC41 | 0.9986394 |
| WRKY50 | ApNAC41 | 0.9986121 |
| WRKY40 | ApNAC41 | 0.9986013 |
| bZIP37 | ApNAC41 | 0.9984386 |
| ERF19 | ApNAC41 | 0.998423 |
| MYB_related41 | ApNAC41 | 0.9982321 |
| WRKY31 | ApNAC41 | 0.9980738 |
| WRKY29 | ApNAC41 | 0.9978049 |
| C2H22 | ApNAC41 | 0.9976602 |
| MYB_related5 | ApNAC41 | 0.9974855 |
| WRKY20 | ApNAC41 | 0.9972835 |
| C2H28 | ApNAC41 | 0.9972585 |
| bZIP13 | ApNAC41 | 0.997166 |
| ERF96 | ApNAC41 | 0.9970102 |
| WRKY4 | ApNAC41 | 0.9967676 |
| WRKY49 | ApNAC41 | 0.9966002 |
| bZIP31 | ApNAC41 | 0.9963245 |
| ERF78 | ApNAC41 | 0.9959976 |
| bZIP14 | ApNAC41 | 0.9949142 |
| MYB_related10 | ApNAC41 | 0.9947042 |
| WRKY34 | ApNAC41 | 0.9946411 |
| bZIP35 | ApNAC41 | 0.9939434 |
| bZIP12 | ApNAC41 | 0.9938544 |
| ERF4 | ApNAC41 | 0.9927774 |
| MYB_related27 | ApNAC41 | 0.9927529 |
| C2H213 | ApNAC41 | 0.9923503 |
| bZIP27 | ApNAC41 | 0.9920336 |
| ERF74 | ApNAC41 | 0.991759 |
| MYB_related12 | ApNAC41 | 0.9907675 |
| WRKY30 | ApNAC41 | 0.9905328 |
| MYB_related4 | ApNAC41 | 0.9905261 |
| MYB_related48 | ApNAC41 | 0.9904555 |
| bZIP5 | ApNAC41 | 0.9901448 |
| bZIP31 | ApNAC06 | 0.9997227 |
| C2H22 | ApNAC06 | 0.9993881 |
| WRKY4 | ApNAC06 | 0.999023 |
| ERF96 | ApNAC06 | 0.9987306 |
| MYB_related1 | ApNAC06 | 0.9986895 |
| MYB_related4 | ApNAC06 | 0.99867 |
| MYB_related5 | ApNAC06 | 0.9984099 |
| ERF19 | ApNAC06 | 0.9982374 |
| MYB_related16 | ApNAC06 | 0.9980088 |
| ERF11 | ApNAC06 | 0.99789 |
| bZIP37 | ApNAC06 | 0.9978548 |
| ERF74 | ApNAC06 | 0.997517 |
| WRKY15 | ApNAC06 | 0.996823 |
| bZIP35 | ApNAC06 | 0.9965499 |
| ERF57 | ApNAC06 | 0.9964068 |
| ERF80 | ApNAC06 | 0.9962139 |
| bZIP10 | ApNAC06 | 0.9959376 |
| WRKY20 | ApNAC06 | 0.9957952 |
| WRKY5 | ApNAC06 | 0.9953479 |
| WRKY50 | ApNAC06 | 0.9948888 |
| WRKY31 | ApNAC06 | 0.9947304 |
| WRKY22 | ApNAC06 | 0.9939512 |
| MYB_related10 | ApNAC06 | 0.9938989 |
| bZIP5 | ApNAC06 | 0.9938301 |
| MYB_related48 | ApNAC06 | 0.9936571 |
| MYB_related41 | ApNAC06 | 0.9932534 |
| WRKY3 | ApNAC06 | 0.9932421 |
| ERF54 | ApNAC06 | 0.9929093 |
| bZIP13 | ApNAC06 | 0.9921228 |
| ERF58 | ApNAC06 | 0.9919082 |
| ERF77 | ApNAC06 | 0.9919076 |
| WRKY29 | ApNAC06 | 0.9918794 |
| bZIP26 | ApNAC06 | 0.9918439 |
| ERF18 | ApNAC06 | 0.9916814 |
| ERF78 | ApNAC06 | 0.9916754 |
| WRKY49 | ApNAC06 | 0.9911628 |
| bZIP43 | ApNAC06 | 0.9908527 |
| MYB_related56 | ApNAC06 | 0.9906046 |
| WRKY23 | ApNAC100 | 0.9984155 |
| WRKY36 | ApNAC100 | 0.9940388 |
| ERF87 | ApNAC05 | 0.9968472 |
| MYB_related49 | ApNAC05 | 0.9952467 |
| WRKY32 | ApNAC05 | 0.9946584 |
| MYB_related12 | ApNAC05 | 0.9911682 |
| bZIP3 | ApNAC05 | 0.9906444 |
| MYB_related27 | ApNAC51 | 0.9992819 |
| WRKY48 | ApNAC51 | 0.9969108 |
| ERF78 | ApNAC51 | 0.9965773 |
| ERF77 | ApNAC51 | 0.9946872 |
| WRKY22 | ApNAC51 | 0.9935183 |
| WRKY42 | ApNAC51 | 0.9935103 |
| MYB_related5 | ApNAC51 | 0.9933991 |
| bZIP20 | ApNAC51 | 0.993098 |
| MYB_related51 | ApNAC51 | 0.9929645 |
| WRKY4 | ApNAC51 | 0.9915951 |
| ERF19 | ApNAC51 | 0.9914951 |
| ERF74 | ApNAC51 | 0.9907677 |
| bZIP14 | ApNAC48 | 0.9980493 |
| C2H28 | ApNAC48 | 0.996487 |
| bZIP12 | ApNAC48 | 0.9960265 |
| WRKY34 | ApNAC48 | 0.9957365 |
| WRKY32 | ApNAC48 | 0.99549 |
| WRKY29 | ApNAC48 | 0.9949235 |
| ERF4 | ApNAC48 | 0.9946519 |
| WRKY49 | ApNAC48 | 0.9945377 |
| bZIP13 | ApNAC48 | 0.9941525 |
| MYB_related12 | ApNAC48 | 0.993753 |
| MYB_related41 | ApNAC48 | 0.992901 |
| WRKY40 | ApNAC48 | 0.9925359 |
| ERF58 | ApNAC48 | 0.9924445 |
| WRKY50 | ApNAC48 | 0.9919054 |
| ERF87 | ApNAC48 | 0.9915083 |
| WRKY31 | ApNAC48 | 0.9913577 |
| ERF8 | ApNAC04 | 0.9994195 |
| WRKY16 | ApNAC04 | 0.9988672 |
| bZIP4 | ApNAC04 | 0.9983178 |
| ERF66 | ApNAC04 | 0.9979242 |
| ERF57 | ApNAC04 | 0.9949325 |
| ERF40 | ApNAC04 | 0.993495 |
| ERF54 | ApNAC04 | 0.992906 |
| ERF80 | ApNAC04 | 0.9927758 |
| ERF41 | ApNAC04 | 0.9904647 |
| WRKY | ApNAC04 | 0.9904346 |
